# Ethanol Impairs Estrogen Receptor Signaling Resulting in Accelerated Activation of Senescence Pathways, Whereas Estradiol Attenuates the Effects of Ethanol in Osteoblasts

**DOI:** 10.1359/jbmr.081011

**Published:** 2008-10-13

**Authors:** Jin-Ran Chen, Oxana P Lazarenko, Rani Lynn Haley, Michael L Blackburn, Thomas M Badger, Martin J Ronis

**Affiliations:** 1 Department of Pharmacology and Toxicology, University of Arkansas for Medical Sciences Little Rock, Arkansas, USA; 2 Arkansas Children's Nutrition Center, University of Arkansas for Medical Sciences Little Rock, Arkansas, USA; 3 Department of Physiology and Biophysics, University of Arkansas for Medical Sciences Little Rock, Arkansas, USA

**Keywords:** alcohol, estrogen receptor, osteoblast, senescence

## Abstract

Epidemiological and animal studies have suggested that chronic alcohol consumption is a major risk factor for osteoporosis. Using bone from cycling female rats infused chronically with ethanol (EtOH) in vivo and osteoblastic cells in vitro, we found that EtOH significantly increased estrogen receptor α (ERα) and β (ERβ) mRNA and ERα protein levels. Treatment with 17β-estradiol (E2) in vivo and in vitro interfered with these effects of EtOH on bone and osteoblastic cells. ERα agonist propylpyrazoletriol (PPT) and ERβ agonist diarylpropionitrile (DPN) attenuated EtOH-induced ERα and ERβ gene overexpression, respectively. Similar to the ER antagonist ICI 182780, EtOH blocked nuclear translocation of ERα-ECFP in the presence of E2 in UMR-106 osteoblastic cells. EtOH also downregulated ERE-luc reporter activity. On the other hand, EtOH by itself upregulated some common ERα- and ERβ-mediated genes apparently by an ER-independent pathway. EtOH also transactivated the luciferase activity of the p21 promoter region independent of additional exogenous ERα, activated p21 and p53, and stimulated senescence-associated β-galactosidase activity in rat stromal osteoblasts. E2 treatment attenuated these EtOH actions. We conclude that inhibitory cross-talk between EtOH and E2 in osteoblasts on ERs, p53/p21, and cell senescence provides a pathophysiologic mechanism underlying bone loss and the protective effects of estrogens in alcohol-exposed females.

## INTRODUCTION

Chronic alcohol abuse has long been recognized as a major risk factor for development of osteoporosis.(1–3) Recent evidence showed binge alcohol exposure can cause bone loss as well.(4) Although the exact mechanism by which ethanol (EtOH) influences bone physiology is not clear, it is likely to involve a combination of both direct and indirect effects on bone cells.(5) EtOH can diffuse into osteoblasts, where alcohol dehydrogenase class 1 (ADH1) is expressed, and be metabolized to acetaldehyde and give rise to reactive oxygen species (ROS) that can exert biological effects.(6) This is considered a direct effect of EtOH. On the other hand, chronic alcohol abuse has been shown to have inflammatory effects in bone marrow, which is known to introduce additional factors that are believed detrimental to the skeleton.(3,7,8) These factors, which include interleukin-1β (IL-1), TNFα, and IL-6, are all secreted by mononuclear cells.(9) EtOH increases RANKL mRNA expression in bone marrow cells, resulting in stimulation of osteoclastogenesis and bone resorption, and this effect was mediated through induction of IL-6.(10) Alcohol consumption also suppresses plasma estrogens in cycling females,(11) suggesting an association between EtOH and reduced estrogen signaling in bone.

Estrogen receptors α (ERα) and β (ERβ) mediate estrogen action in a variety of tissues and cells. Whereas these receptors have overlapping responsibilities, they also have distinct roles in different tissue and cell types.(12) In human osteoblasts where both estrogen receptors are present, genes regulated by estrogen through ERα and/or ERβ show commonalities, but also significant differences.(13) Chronic EtOH ingestion in humans and rats induce an increase in ER expression in liver and breast.(14,15) EtOH-induced increases in mammary ER expression may be associated with an increased risk of breast cancer. However, the significance of EtOH-induced overexpression of ER in other tissues and the consequences for tissue pathology has not been studied. Bone is also recognized as an estrogen sensitive organ, and altered ER signaling in bone cells are known to affect bone quality. Although we have previously predicted that EtOH might alter ER signaling in bone cells,(1) published evidence to support this hypothesis is lacking.

Overexpression of ERs in osteoblasts has been linked to activation of p53.(16) p53 is a well-known tumor suppressor gene that maintains the integrity of the genome by its ability to regulate various aspects of cell growth and apoptosis.(17) p53 is also believed to represent a key component of a signal transduction pathway that is stimulated by increased intracellular ROS to trigger apoptosis, and p53 activation also results in ROS generation in the mitochondria.(18–20) Studies analyzing the role of p53 in different cell types have shown a tissue-specific role for p53 in differentiation of several tissue types.(21,22) p53 has been shown to regulate osteoblast differentiation, bone formation, and osteoblast-dependent osteoclast differentiation.(23) Activation of p53 in bone tissue has been observed both in aged animals and in sex steroid-deficient gonadectomized mice.(24) Activated p53 has been suggested to be a link to the senescence pathway in bone cells.(16) Moreover, EtOH has recently been reported to induce p53.(25,26) p21 functions as a downstream effector of p53, and previous studies have implicated p21 involvement in ER signaling.(27) These findings suggest that impaired estrogen signaling in bone cells after EtOH exposure could accelerate senescence

In the studies presented in this report, we attempted to understand the effect of EtOH treatment in vivo and in vitro on estrogen receptors and ER signaling compared with classic ER ligands, such as 17β-estradiol (E2) and ICI in osteoblasts. We also tried to determine whether EtOH activates p53 and p21 and its promoter and if this led to accelerated senescence in osteoblasts. We present a new paradigm whereby EtOH induces bone loss by impairing ER signaling and the interaction between ER and p21 to determine the fate of osteoblasts. These data also provide a further explanation for why endogenous or exogenous estrogens antagonize the toxic effects of EtOH on bone.

## MATERIALS AND METHODS

### Animals

Female Sprague-Dawley rats (250–260 g) were purchased from Charles River Laboratories (Wilmington, MA, USA). Animals were housed in an Association for the Assessment and Accreditation of Laboratory Animal Care-approved animal facility. Animal maintenance and experimental treatments were conducted in accordance with the ethical guidelines for animal research established and approved by the Institutional Animal Care and Use Committee at University of Arkansas for Medical Science (Little Rock, AR, USA). Rats were surgically implanted with an intragastric cannula as described previously,(28,29) and rats were fed by total enteral nutrition (TEN). Liquid diets were formulated to contain the nutrients recommended for rats by the National Research Council. The TEN animal model has been detailed previously.(1) Diets contained 16% protein, 54% carbohydrate, and 25% fat (corn oil), and EtOH-containing diets were kept isocaloric to the control diets by substituting EtOH for carbohydrate calories. The EtOH dose was 12 g/kg/d. Rats (*n* = 8/group) were infused 187 kcal/kg^3/4^/d for 14 h from 6:00 p.m. to 8:00 a.m. during the dark cycle for 4 wk. Additional groups of control and EtOH-infused animals were supplemented with subcutaneous E2 (20 μg/kg/d; Sigma-Aldrich, St Louis, MO, USA) administered using Alzet osmotic minipumps.(1) All rats were weighed every other day, and we found that there are no significant differences among all four groups in body weight in the end of experiment: control, 278.1 ± 1.8 g; EtOH, 273.8 ± 2.1 g; E2, 278.1 ± 2.3 g; E2 + EtOH, 280.2 ± 3.7 g.

### Cell culture

Control cycling female rat bone marrow cells were harvested from femurs according to methods described previously.(30) To have stromal osteoblasts for treatment, bone marrow cells were seeded at a density of 3 × 10^6^ cells/well in six-well cell culture plates in the presence of MEM (Invitrogen, Carlsbad, CA, USA) with 10% FBS (Hyclone Laboratories, Logan, UT, USA) and 1 mM of ascorbyl-2-phosphate (Sigma-Aldrich), 4 mM l-glutamine, and 100 U/ml of each penicillin and streptomycin (Sigma-Aldrich), conditions known to drive osteoblast differentiation. One half the cell culture medium was changed every 5 days, and after 20 days, mature osteoblasts were developed for treatment. Neonatal rat calvarial osteoblastic cells were isolated from untreated 4-day-old rat pups by sequential collagenase digestion using a method described previously.(31) Rat calvarial osteoblastic cells and the rat osteoblast-like cell line UMR-106 (ATCC, Rockville, MD, USA) were cultured in αMEM supplemented with 10% FBS. When cells were ready to be treated, culture medium was saturated with O_2_ and CO_2_ in an incubator for 2 h and plates were sealed during EtOH treatment; these treatment procedures were detailed previously.(6)

### Real-time RT-PCR analysis

Rat tibial bone RNA and osteoblastic cell RNA were extracted using TRI Reagent (MRC, Cincinnati, OH, USA) according to the manufacturer's recommendation followed by DNase digestion and column clean-up using QIAGEN minicolumns. Briefly, at the time of death, the right tibia was taken, and bone marrow cells were flushed with Eagle's MEM + Hanks' salts after cleaning the surrounding connective tissue. Tibial bones were frozen in liquid nitrogen. Tibial bone was placed in 1000 μl TRI Reagent and homogenized using a polytron-aggregate (Kinematica). One hundred microliters of 1-bromo-3-chloropropane (BCP) was added, and the mixture was centrifuged for 15 min at speed of 16,000 rpm and 4°C. Four hundred fifty microliters supernatant was taken, and an equal volume of isopropanol was added and centrifuged for additional 15 min (16,000 rpm, 4°C). After washing the RNA pellet with 75% ethanol, isolated RNA was resuspended in RNase-free water. Treated cells from 6-well plates were washed with twice with PBS, and 1000 μl TRI Reagent was added into each well. Cells were scraped into a 1.5-ml Eppendorf tube. RNA preparation was identical to that of isolation of RNA from bone tissue. Reverse transcription was carried out using an iScript cDNA synthesis kit from Bio-Rad (Hercules, CA, USA). Real-time RT-PCR was carried out using SYBR Green and an ABI 7000 sequence detection system (Applied Biosystems, Foster City, CA, USA). Primers for rat ERα and ERβ, PTH-like hormone (PTHLH), bone morphogenetic protein 6 (BMP6), phosphodiesterase 3B (PDE3B), Bcl-2-associated transcription factor (BTF), Autotaxin, and stromal antigen 2 (STAG2) were designed using Primer Express software 2.0.0 (Applied Biosystems), and all primer sequences used in this study are listed in Table 1.

**Table 1:** Real-Time RT-PCR Primer Sequences

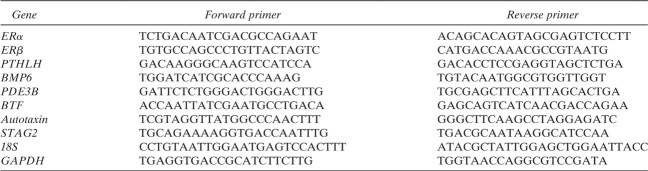

### Western blotting

Tibia bone tissue proteins and in vitro cellular proteins were extracted using a cell lysate buffer as described previously.(6) Phosphorylation of p53 and total p53 in bone tissue and in vitro osteoblasts was assessed by Western immunoblotting using goat polyclonal antibody recognizing phosphorylated p53 (Santa Cruz Biotechnology) and rabbit polyclonal antibody recognizing total p53 (Cell Signaling), followed by incubation with either an anti-goat or an anti-rabbit antibody conjugated with horseradish peroxidase (Santa Cruz). The status of p21, ERα, and GAPDH protein in bone tissue and in vitro osteoblasts were analyzed by immunoblotting, using rabbit polyclonal antibodies recognizing p21 and ERα (Santa Cruz) and a mouse monoclonal antibody recognizing GAPDH (Santa Cruz), followed by incubation with either an anti-rabbit or an anti-mouse antibody conjugated with horseradish peroxidase (Santa Cruz) and SuperSignal West Pico chemiluminescent substrate (Pierce). Quantitation of the intensity of the bands in the autoradiograms was performed using a VersaDoc imaging system (Bio-Rad).

### DNA constructs and luciferase activity assays

ERα transcriptional activity was determined by measuring the EtOH- and estradiol-stimulated, ERα-mediated activation of the estrogen-responsive reporter plasmid ERE-TK-Luc. The ERE-TK-Luc reporter and wildtype ERα plasmid used in this report were published previously.(32) Human p21 promoter pGL2-p21-Luc, containing the p21 promoter-luciferase reporter fusion and two p53-binding consensus sites, was kindly provided by Dr Lieberman.(33) ERE-TK-Luc and pGL2-p21-Luc plasmids were transfected into UMR-106 cells with or without ERα co-transfection using Lipofectamine 2000 (Invitrogen). Transfected cells were treated with E2, ICI 182780 (Faslodex) and EtOH for 24 h, and lysates were harvested for luciferase assays. The experiment was performed according to protocols from the manufacturer (Promega). Luciferase activity was measured on a MLX Microtiter Plate Luminometer (Dynex Technologies, Chantilly, VA, USA).

### Transient transfection and subcellular localization of ERα

Using 24-well plates, UMR-106 cells were transiently transfected using Lipofectamine 2000 (Invitrogen), with full-length wildtype ERα inserted into the appropriate ECFP vector (Clontech), along with red fluorescent protein (pDs 1Red-N1; Clontech, Palo Alto, CA, USA) targeted to the nucleus (nRFP). These two constructs were detailed previously.(32) Transfected cells were cultured for 24 h. Subsequently, cells were serum-starved by culturing in the presence of 2% BSA for 4 h and treated with vehicle, 50 mM EtOH, 10^−9^ M E2, and 10^−7^ M ICI 182780 for 2 h. The cells showing either nuclear or cytoplasmic accumulation of ERα were directly visualized using a fluorescence microscopy with connection to a camera.

### Senescence-associated β-galactosidase activity and staining

Fully differentiated rat bone marrow stromal osteoblasts in 6- and 24-well plates were treated with E2 for 30 min before adding 50 mM EtOH, and plates were well sealed for 24 and 48 h. The senescence-associated β-galactosidase activity assay, performed using the β-galactosidase enzyme assay kit (Promega), measured the absorbance at 420 nm according to the manufacturer's instruction. Cell β-galactosidase staining was also performed according to a method published previously.(34) Senescent cells were identified as blue-stained cells by standard light microscopy.

### Statistical analyses

Data are expressed as means ± SD. One-way and two-way ANOVA followed by Student-Newman-Keuls posthoc analysis was used to compare the treatment groups. Values were considered statistically significant at *p* < 0.05.

## RESULTS

### EtOH stimulates estrogen receptor overexpression and activation of p53 and p21 in bone tissue

**FIG. 1:**
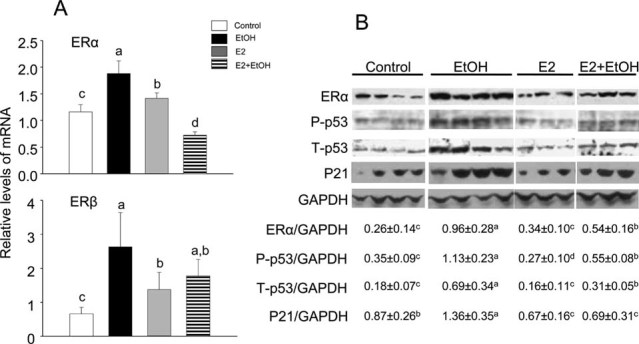
EtOH induces estrogen receptor expression and activation of p53 and its downstream effector p21 in bone. Tibial bone RNA and proteins (*n* = 7/group) were isolated. (A) Real-time PCR was performed for ERα and ERβ gene expression, and the volumes were normalized to GAPDH control gene expression. (B) Western blots of ERα, p21, phospho-p53, total p53, and GAPDH are depicted for four samples from control and EtOH groups and three samples from E2 and E2 + EtOH groups. Numbers represent means ± SD of the ratios of intensity of the bands of each target proteins over GAPDH protein for each treatment. Bars represent means ± SD for *n* = 7 animals/group. Means with different letters differ significantly from each other at *p* < 0.05, a > b > c > d as determined by two-way ANOVA followed by Student-Newman-Keuls posthoc analysis for multiple pairwise comparisons.

Using pQCT, we found that, in the EtOH-infused group, trabecular BMD was significantly lower compared with the control total enteral nutrition group (control: 234.6 ± 11 versus EtOH: 182.7 ± 8.2 mg/cm^3^, *n* = 7; *p* < 0.05). E2 treatment (E2: 300.0 ± 11.2 mg/cm^3^, *n* = 7) increased trabecular BMD (*p* < 0.05 versus control) and reversed the effect of EtOH on tibial trabecular BMD (E2 + EtOH: 284.8 ± 8.3 mg/cm^3^, *n* = 7; *p* < 0.05 versus EtOH). These results were similar to those we have previously published.(1) Using RNA extracted from tibial bone, real-time PCR was carried out for estrogen receptor subtypes α and β (ERα, ERβ). EtOH and E2 treatment both upregulated ERα and ERβ gene expression (*p* < 0.05), although EtOH had a larger effect (Fig. 1A). Interestingly, in the E2 + EtOH group, E2 attenuated EtOH effects on both ERα and ERβ gene expression. Similarly, this EtOH and E2 interaction was observed when ERα protein levels were evaluated using Western blot (Fig. 1B). Because previous evidence indicated that EtOH can increase p53 expression in heart tissue,(35) we measured p53 and its phosphorylation status along with p21 levels. Both total and phosphorylated p53 were increased in the EtOH group, and E2 attenuated these effects (Fig. 1B). The p21 protein expression pattern in bone was similar to that of p53 protein expression in the EtOH, E2, and EtOH + E2 groups (Fig. 1B).

### E2 and EtOH regulate common, ERα-, and ERβ-specific mediated genes in bone

**FIG. 2:**
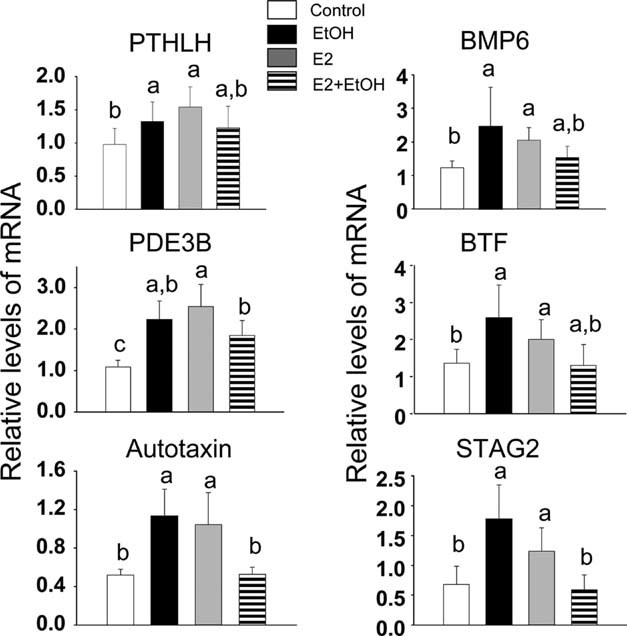
Interactions of EtOH and E2 on ERα- and ERβ-mediated specific gene expression in bone. Real-time PCR was carried out for six different ERα and ERβ regulated genes using RNA isolated from tibial bone. mRNA expression was normalized to control GAPDH gene expression, (*n* = 7/group). Bars represent means ± SD for *n* = 8–10 animals/group. Means with different letters differ significantly (*p* < 0.05, a > b > c > d) as determined by two-way ANOVA followed by Student-Newman-Keuls posthoc analysis for multiple pairwise comparisons.

Prompted by the above data that EtOH upregulates ERα and ERβ gene expression in bone, we next studied EtOH and E2 cross-talk through ERα and ERβ. The following six genes were selected: *PTHLH*, *BMP6*, *PDE3B*, *BTF*, *Autotaxin*, and *STAG2*. According to data published by Stossi et al.,(13) *PTHLH* and *BMP6* are considered common genes mediated through ERα and ERβ, *PDE3B* and *BTF* are mediated specifically through ERα, whereas *Autotaxin* and *STAG2* are mediated through ERβ specifically. Using RNA extracted from tibial bone and real-time PCR analysis, we found that EtOH and E2 alone upregulated all six genes with no specificities for either ERα or ERβ. Interestingly, in the EtOH + E2 group, all six gene expressions were lower (*p* < 0.05) compared with EtOH or E2 alone, especially *Autotaxin* and *STAG2*, which were lowered back to control level (Fig. 2).

### E2 interacts with EtOH to regulate ERα and ERβ gene expression in in vitro osteoblastic cell cultures

**FIG. 3:**
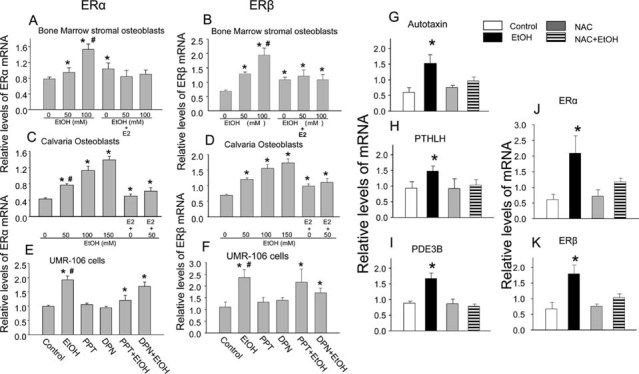
EtOH upregulates estrogen receptors and interacts with their ligands in vitro. Osteoblasts from three different sources were used for identifying the action of EtOH on estrogen receptor gene expression and characterization of interactions with estrogen receptor ligands. (A and B) Stromal osteoblastic cells cultured from control rat bone marrow cells were treated with EtOH at 50 and 100 mM in the presence or absence of 10^−9^ M E2 (30-min pretreatment) for 24 h. (C and D) Isolated neonatal calvarial osteoblasts were treated with EtOH at 50, 100, and 150 mM. 50 mM EtOH-treated wells were in the presence or absence of 10^−9^ M E2 (30-min pretreatment). (E and F) UMR-106 osteoblastic cells were treated with 50 mM EtOH in the presence or absence of 10^−8^ M ERα-specific agonist PPT or ERβ-specific agonist DPN (30-min pretreatment) for 24 h. RNA was isolated, and ERα and ERβ mRNA was measured by real-time RT-PCR. All mRNA expressions were normalized to GAPDH mRNA expression. (G-K) UMR-106 osteoblastic cells were pretreated with 10 mM NAC for 30 min before adding 50 mM EtOH. After 24-h treatment, cell RNA was extracted. Real-time PCR was performed for evaluation of indicated gene expression. Bars represent means ± SD. *p < 0.05 vs. control vehicle-treated cells. ^#^*p* < 0.05 EtOH vs. E2 + EtOH in A and B, EtOH vs. PPT + EtOH in E, and EtOH vs. DPN + EtOH in F in 6-well plate triplicates as determined by two-way ANOVA followed by Student-Newman-Keuls posthoc analysis for multiple pairwise comparisons.

To further confirm the interaction of E2 and EtOH on ERα and ERβ gene expression specifically in osteoblasts, we used stromal osteoblasts derived from bone marrow cells, calvarial osteoblasts isolated from neonatal calvaria, and the UMR-106 osteoblastic cell line. In the first set of experiments, mature stromal osteoblasts derived from bone marrow were treated with EtOH using 50 and 100 mM, with or without 30-min 10^−9^ M E2 pretreatment. Twenty-four hours later, RNA was extracted, and real-time PCR and ERα and ERβ gene expression were studied. EtOH induced ERα and ERβ gene expressions in a concentration-dependent fashion. In agreement with the data we obtained in vivo, E2 induced both ERα and ERβ gene overexpression. As we expected, E2 did not show an additive effect but rather attenuated EtOH's effect on both ERα and ERβ gene expression at two different EtOH concentrations (Figs. 3A and 3B). Similarly, in the second set of experiments, concentration-dependent responses of ER gene expression to EtOH were observed using isolated neonatal calvarial osteoblasts, and E2 was also able to attenuate 50 mM EtOH-induced expression of both ER genes (Figs. 3C and 3D). To test the hypothesis that the action of EtOH on ER gene expression is non-receptor specific, we used ERα specific agonists PPT and ERβ specific agonist DPN to treat UMR-106 osteoblastic cells with or without EtOH. We found that PPT and DPN were able to attenuate EtOH-induced ERα and ERβ gene expression specifically (Figs. 3E and 3F). This suggested that EtOH stimulates ER gene expression in a nonselective fashion but that ERα- and ERβ-specific pathways are involved in feedback regulation of each receptor type. To test the hypothesis that EtOH activates some common ER-regulated genes because of activated oxidative stress in osteoblasts, we used UMR-106 osteoblastic cells and treated the cells with *N*-acetyl cysteine (NAC), which is a well-known antioxidant chemical together with EtOH. We found that pretreatment with 10 mM NAC not only attenuated EtOH-induced ERα and ERβ gene expression but also completely blocked EtOH-activated ER-regulated (common, ERα specific and ERβ specific) gene expression in those osteoblasts (Figs. 3G and 3K).

### Differential effects of EtOH and E2 on ERE and p21 promoter reporter in UMR-106 osteoblastic cells

**FIG. 4:**
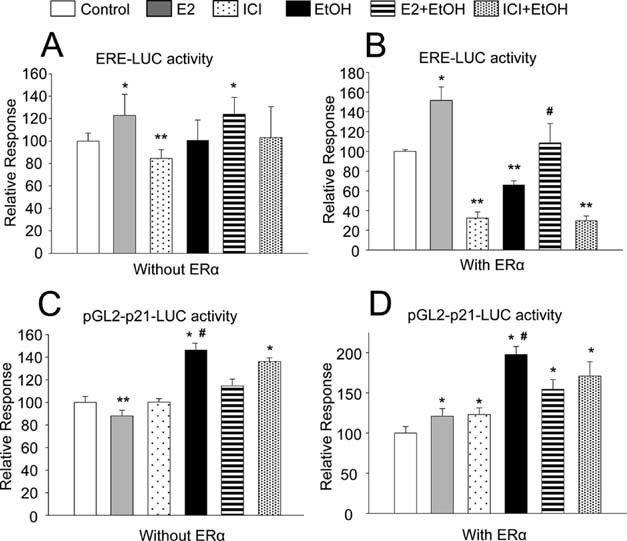
E2, but not ICI, alters EtOH-induced regulation of ERE-TK-Luc and p21 promoter activities in osteoblasts. (A and B) Rat UMR-106 osteoblastic cells in 24-plate quadruplicates were transfected with ERE-TK-Luc plasmid with or without co-transfection of wildtype ERα. (C and D) Rat UMR-106 osteoblastic cells in 24-plate quadruplicates were transfected with pGL2-p21-Luc plasmid with or without co-trnasfection of wildtype ERα. Twenty-four hours later after transfection, cells were treated with EtOH 50 mM, E2 10^−9^ M, ICI 10^−7^ M, and a combination of E2 + EtOH and E2 + ICI (with E2 10^−9^ M and ICI 10^−7^ M 30-min pretreatment before adding EtOH) for an additional 24 h. Luciferase activity was measured. All data were corrected for renilla activity and relative to control treated with or without ERα, respectively. *Significantly greater and **significantly lower than control vehicle treated cells in 24-well plate quadruplicates at *p* < 0.05. ^#^Significantly different when EtOH and E2 + EtOH were compared at *p* < 0.05 as determined by two-way ANOVA followed by Student-Newman-Keuls posthoc analysis for multiple pairwise comparisons.

Based on the above observations that ER genes and p21 expression are induced by EtOH, we next compared the effect of EtOH, E2, and ICI on the estrogen-responsive reporter and the p21 promoter. ERE-TK-Luc or the human pGL2-p21-Luc was transfected into UMR-106 osteoblastic cells, with or without co-transfection with ERα. Transfected cells were treated with EtOH, E2, and ICI and their combinations for 24 h. The cells were harvested and assayed for luciferase activity. ERE-TK-Luc activities were increased by up to 50% and 20% in response to E2 with or without ERα, respectively. Interestingly, similar to ICI, EtOH decreased ERE-TK-Luc activity by 40% in the presence of ERα. However, EtOH had no effect without exogenous ERα (Figs. 4A and 4B). In the absence of exogenous ERα, the combination of E2 and EtOH increased ERE-TK-Luc activity similar to that of E2 alone. In the presence of exogenous ERα, the combination of EtOH and E2 returned ERE-TK-Luc activity back to control levels. In the combination EtOH and ICI treatment, ERE-TK-Luc activity was decreased even further compared with EtOH alone (Figs. 4A and 4B). EtOH had an opposite effect on pGL2-p21-Luc activity compared with its effect on ERE-TK-Luc activity. Whether exogenous ERα was present or not, EtOH activated pGL2-p21-Luc activity (Figs. 4C and 4D). E2 not only decreased p21 luciferase activity on its own, but also attenuated EtOH-induced activity in the absence of exogenous ERα (Figs. 4C and 4D). On the other hand, ICI had no effect on p21 luciferase activity by itself, and it had no effect on EtOH-induced activity in the absence of exogenous ERα. In the presence of ERα, both E2 and ICI slightly activated pGL2-p21-Luc activity, and they both lowered EtOH's activation of pGL2-p21-Luc when combined with EtOH, with E2 having a greater effect than ICI (Figs. 4C and 4D).

### Cellular localization of ERα in UMR-106 cells

**FIG. 5:**
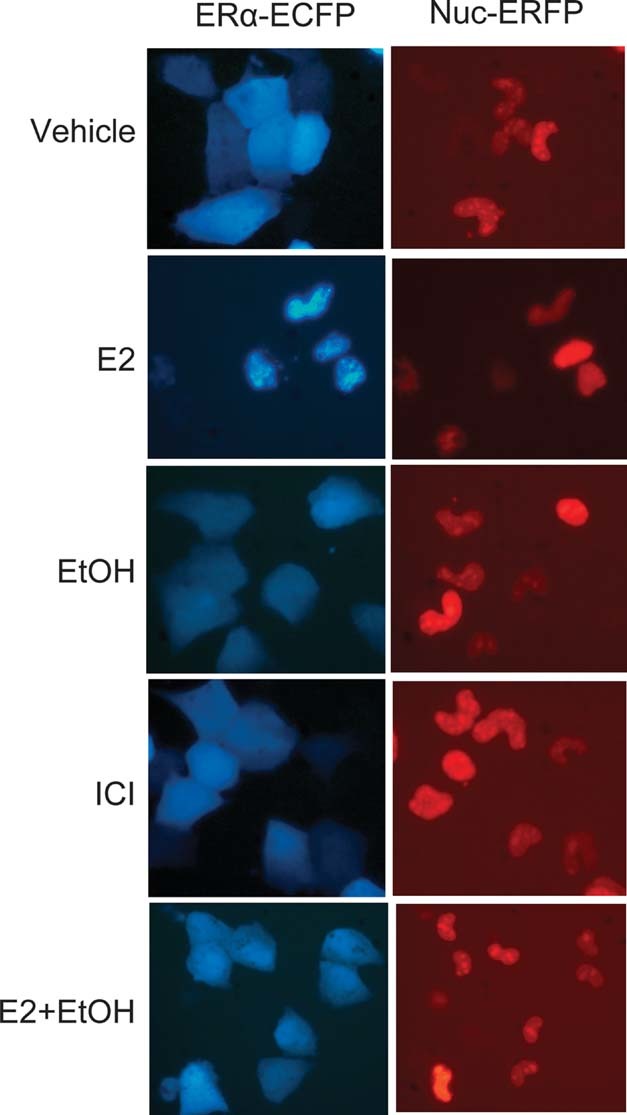
Cellular localization of ERα in UMR-106 cells. UMR-106 osteoblastic cells were grown in a 24-well plate, transfected with an ERα-ECFG expression plasmid (0.4 μg/well) together with nRFP nuclear anchor protein (0.1 μg/well), and treated with vehicle, 50 mM EtOH, 10^−9^ M E2, and 10^−7^ M ICI for 2 h before viewing (×20 lens) directly under a fluorescent microscopy connected with a camera. In EtOH and E2 combination treatment (E2 + EtOH), E2 was added 30 min before EtOH treatment. In the EtOH-, E2 + EtOH–, and ICI-treated cells, ERα-ECFG was found throughout the cell, whereas in E2-treated cells, ERα-ECFG translocated into the nucleus. Pictures were taken under fluorescent microscopy (×20) from representative areas from each treatment well.

To explore the differences of the effects of EtOH and E2 on estrogen receptor in bone cells, we compared the cellular distribution of ERα-ECFP expression plasmid together with Nuc-ERFP in UMR-106 osteoblastic cells by fluorescent microscopy. After ERα-ECFP was transfected into a cell, ERα was distributed mainly in the cytoplasm of control treated cells. The addition of E2 resulted in rapid nuclear translocation of ERα, whereas ERα from cells treated with EtOH or ICI remained in the cytoplasm (Fig. 5). Like ICI, we found that the majority of ERα-ECFP was in the cytosol after addition of EtOH to E2-treated cells for 2 h (Fig. 5).

### Senescence-associated β-galactosidase activity is increased in EtOH-treated osteoblasts

**FIG. 6:**
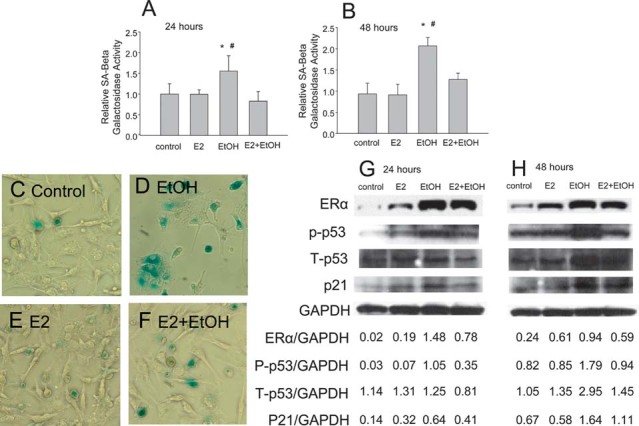
EtOH induces ERα, activates p53 and p21, and increases senescence-associated β-galactosidase activities in bone marrow stromal osteoblasts. Mature stromal osteoblasts derived from bone marrow cells in the presence of osteogenic medium were treated with EtOH, E2, and E2 + EtOH for 24 and 48 h. (A and B) Senescence-associated β-galactosidase enzyme activity measured from cell lysates collected 24 and 48 h after treatment. EtOH increased enzyme activity at 24 and 48 h; however, E2 blocked or attenuated the effects of EtOH at either 24 or 48 h. (C-F) Senescence-associated β-galactosidase cell staining was performed after 48-h treatment. Blue stained cells showed positive for senescence-associated β-galactosidase activity. (G and H) Western blots showed increased ERα, p53, and p21 and increased phosphorylation of p53 by EtOH in cell lysates collected at 24 and 48 h. E2, however, attenuated the effects of EtOH. The numbers represent the ratio of intensity of the band of targeted protein over control GAPDH for a single treatment. Bars represent means ± SD. *p < 0.05 vs. control vehicle treated cells in 6-well plate triplicates. ^#^Significantly different EtOH vs. E2 + EtOH (*p* < 0.05 as determined by two-way ANOVA followed by Student-Newman-Keuls posthoc analysis for multiple pairwise comparisons).

Previous reports have strikingly shown that an activating mutation of p53 causes early onset of aging-associated phenotypes in mice.(36) Our above data showed that EtOH induces p53 and p21 in bone. This further prompted us to examine senescence-associated β-galactosidase activity in osteoblasts treated with EtOH and E2. Mature stromal osteoblasts were exposed to EtOH and E2 for 24 and 48 h. β-galactosidase activity was increased 50% in EtOH-treated cells after 24 h and >100% after 48 h (Figs. 6A and 6B). E2 completely blocked the effect of EtOH after 24 h but only attenuated the effect after 48-h treatment. Consistently, EtOH-treated cells showed more β-galactosidase positive blue cells after 48 h (Figs. 6C and 6F). Cell lysates were collected after 24- and 48-h treatment for Western blot analysis of ERα, p53, and p21 protein expression. Similarly to what we found in bone tissue in vivo (Fig. 1B), EtOH activated p53 and p21, whereas E2 attenuated these effects (Figs. 6G and 6H).

## DISCUSSION

In human alcoholics and in experimental animal studies, EtOH has been shown to cause a variety of tissue damage. For example, EtOH-induced liver damage resulting from overproduction of reactive oxygen species (ROS) has been well documented.(37) We have previously developed a female rat model in which EtOH is infused overnight as part of a system of TEN to mimic consumption patterns and blood EtOH concentrations observed in alcoholics.(1,2) Using this model, we showed that bone loss occurred after chronic EtOH infusion independent of nutritional status.(1,2) In addition, we showed that EtOH treatment reduces estradiol in cycling females and disrupts estrogen or ER signaling in bone.(1,6) In this report for the first time, we showed that EtOH also directly stimulates overexpression of ERs in bone in vivo and in osteoblasts in vitro and that this can be reversed by ER agonist treatment. In general, ERs are ligand-inducible transcription factors. When bound to E2, ERs activate the expression of genes that have estrogen responsive elements (EREs) in their promoter regions. EtOH, which is not a ligand of ER, stimulated both receptors to be overexpressed but also prevented E2-stimulated ERα translocation to the nucleus and downregulated ERE-luc reporter activity in osteoblasts. These data suggest that the ability of EtOH to upregulate ER expression is a feedback consequence of impaired ER signaling. In agreement with our present data, a previous study reported that ER concentration is increased in hepatocytes after EtOH stimulation and suggested that EtOH administration could be considered as a form of chemical castration.(38) However, these data are in contrast to previously published data in human breast cancer cell lines in which EtOH and E2 synergistically activated ERE-luc reporter activity.(15) This may suggest tissue-specific interactions between EtOH and ER signaling.

Bone tissue is known to express both ERs, unlike other tissues such as uterus, liver, ovary, and prostate, where one of the two ERs predominates.(39) We attempted to find differences between ERα- and ERβ-regulated gene expression in osteoblasts in response to EtOH stimulation. By far, the most extensive previous evidence on ER-specific signaling in osteoblasts has been recently published by Stossi et al.(13) They showed that, in human osteoblastic cells, *BMP6* and *PTHLH* are ERα and ERβ common, *PDE3B* and *BTF* are ERα specific, and *ENPP2* and *STAG2* are ERβ-specific genes in response to E2.(13) We found that E2 induced expression of all of these genes but that the combination of E2 and EtOH attenuated this response. These data are also consistent with the hypothesis that EtOH impairs ER-mediated signaling in osteoblasts.

An interesting finding in this study was that the p21 promoter in osteoblasts is activated by EtOH in the presence or absence of exogenous ERα and that p21 protein expression was significantly increased. This was accompanied by increased phosphorylation of p53. The biological significance of these findings is that p53 and p21 have previously been found to be accumulated in alcoholic tissues and associated with cancer occurrence.(40) In normal physiologic situations, in breast cancer cells, ER and p53 exert opposing effects on cellular proliferation. Recent evidence has shown that ER may directly bind to p53, leading to downregulation of transcriptional activation by p53.(41) There is a lack of extensive evidence on the relationship between ER and p53 in osteoblasts and how estrogen regulates p53 is also controversial.(42) β-galactosidase is well established as a biomarker to identify senescent cells in culture and aging skin in vivo.(34) The increase in p21 expression and p53 activity after EtOH treatment was associated with higher levels of SA-β-galactosidase activity. This suggests that the senescence pathway is activated by EtOH in these osteoblasts. Our data showed that co-treatment of EtOH-treated osteoblasts with E2 resulted in a reversal of effects on p21 promoter activation, p21 overexpression, and p53 phosphorylation and inhibition of EtOH-induced senescence. These data are consistent with our previous studies that have shown that E2 treatment can prevent EtOH-induced bone loss in female rats(1,6) and suggest negative cross-talk between ER-mediated signaling and EtOH-stimulated cell signaling.

**FIG. 7:**
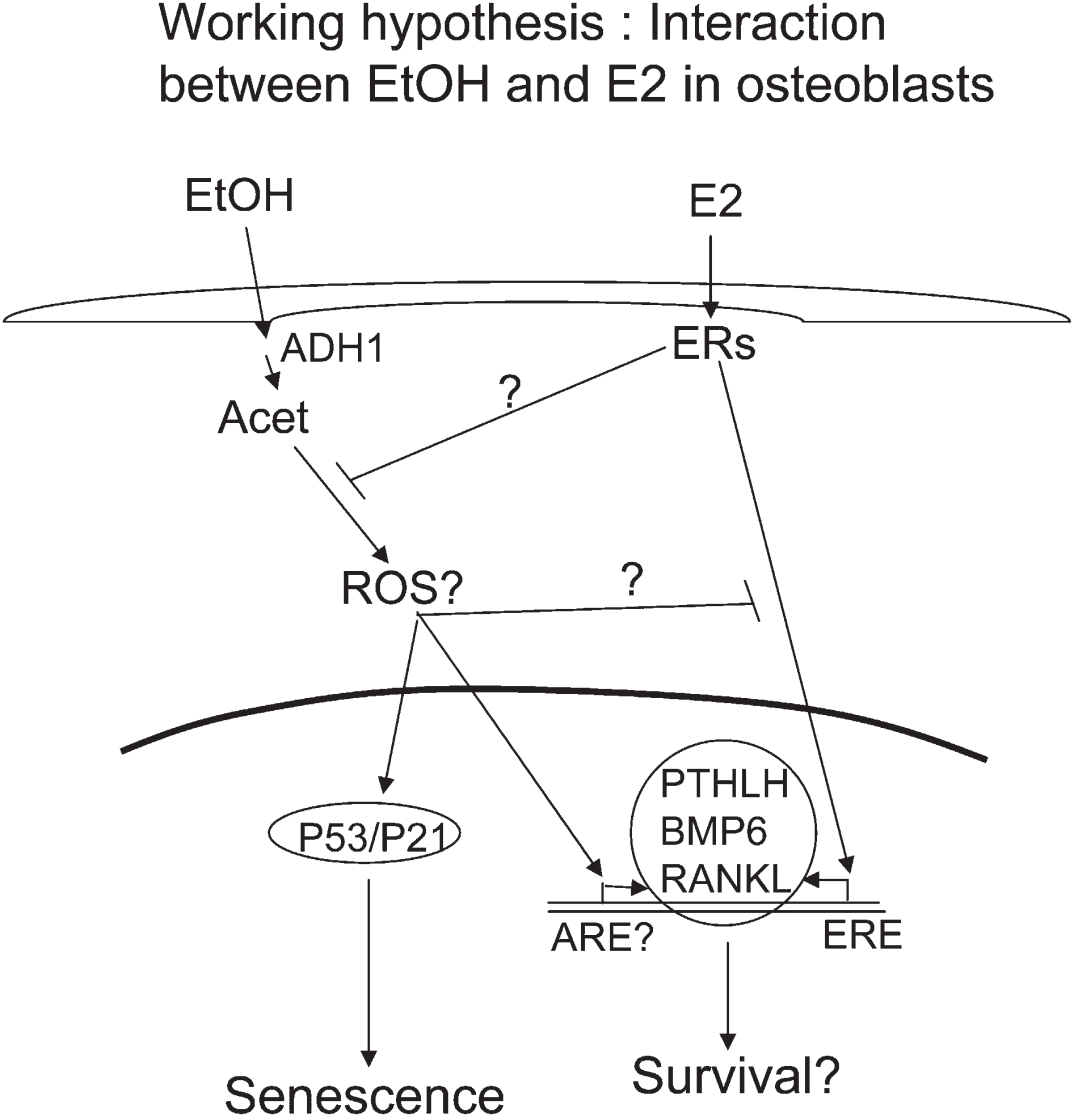
Working hypothesis describing interactions between EtOH and E2 in osteoblasts. Estrogens act on osteoblast through their receptors (ERs) and ER is translocated into the nucleus where it activates estrogen responsive elements (ERE), leading to altered gene expression promoting osteoblast survival. On the other hand, EtOH diffuses cells where it is metabolized to acetaldehyde (Acet) by alcohol dehydrogenase class I (ADH1) and also gives rise to reactive oxygen species (ROS), which can exert several biological effects on the cell: (1) it may activate the p53/p21 complex, (2) it may activate AREs, which have not been identified, and/or (3) it may also block the ER nuclear translocation. These observations suggest the existence of EtOH and E2 cross-talk in osteoblasts that can alter cell fate.

In this regard, the one surprising finding of this study was that the six ER-regulated genes we chose to study—*BMP6, PTHLH, PDE3B, BTF, ENPP2,* and *STAG2*—were all expressed at higher levels after EtOH treatment alone in addition to being induced by E2 but that the combination of EtOH and E2 led to reduced expression. The most likely explanation for this apparently contradictory finding is that EtOH induces these genes through an ER-independent pathway probably involving increased production of ROS in osteoblasts(6) and that negative cross-talk of E2 on EtOH signaling and of EtOH on ER signaling explains the effects of EtOH + E2 (Fig. 7). Another less likely possibility is that, although EtOH inhibits ER-mediated signaling through ERE elements, it could stimulate some ER-mediated signaling pathways through indirect effects on ER interactions with transcription factors at other elements such as AP1 or Sp1 sites.(43–45) In this regard, it should be pointed out that, as with p21 in this study, in our previous studies with RANKL in osteoblasts, E2 treatment either has no effect or suppresses RANKL mRNA, EtOH induces RANKL mRNA expression, and the combination of E2 and EtOH prevents this induction.(1,6) In addition, osteoblasts treated with the antioxidant NAC together with EtOH showed that NAC completely blocked EtOH-induced gene expression. These observations further suggested that ROS is involved in the negative cross-talk of E2 on EtOH signaling in osteoblasts. However, whether the putative ER membrane (versus nuclear) pathway is responsible for this effect clearly needs to be investigated in future studies. Moreover, G protein-coupled receptors, which have currently been shown to directly bind estrogen,(46) may be the other possible mechanism for the effect of EtOH in osteoblasts.

From our data, as summarized in Fig. 7, we believe that EtOH-triggered accelerated osteoblastic cell senescence results from activation of p53 and p21. This action of EtOH and negative cross-talk between EtOH signaling and ER-mediated pathways contributes to osteoporotic bone loss in chronic alcohol drinkers and protective effects of estrogens on EtOH-induced bone loss.
